# A comprehensive workflow for CCTA and OCT data fusion with 3D printing validation: advancing patient-specific testing environments for percutaneous coronary intervention devices

**DOI:** 10.1186/s12938-025-01501-6

**Published:** 2025-12-23

**Authors:** Marc Ilic, Jonas Häner, Julian Lehmann, Anselm W. Stark, Joël Illi, Christoph Gräni, Marc Bentele, Philine Baumann-Zumstein, Julia Busch, Andreas Haeberlin

**Affiliations:** 1 Department of Cardiology, University Hospital Bern, Freiburgstrasse 20, CH-3010 Bern, Bern Switzerland; 2 R&D, Biotronik AG (a Teleflex company), Ackerstrasse 6, CH-8180 Bülach, Zürich Switzerland; 3 Medical Affairs, Biotronik AG (a Teleflex company), Ackerstrasse 6, CH-8180 Bülach, Zürich Switzerland

**Keywords:** Cardiovascular, Coronary artery, Digital model, Oct, Phantoms

## Abstract

**Objective:**

To create high-resolution, patient-specific 3D coronary artery models aimed at developing digital models and functional phantoms for the testing of cardiac catheterization devices.

**Methods:**

Using coronary computed tomography angiography (CCTA) and optical coherence tomography (OCT), coronary artery lesions were identified and quantified. Imaging data were fused using a custom-made workflow to create highly accurate digital 3D models. For validation of the workflow, coronary artery phantoms were fabricated using additive manufacturing. An OCT was then conducted on the 3D printed phantom, and the developed workflow was applied to generate a derivative model, which was subsequently compared to the original.

**Results:**

CCTA and OCT datasets from 15 patients were successfully collected and used to develop patient-specific 3D coronary artery models, including detailed inner shells, calcifications, outer wall structures, and side branches. Of these, 13 out of 15 3D printed phantoms were successfully validated and compared to their corresponding original model. The median vertex deviation of the derivative model was 0.15 (0.14$-$0.17) mm. The median absolute stenosis difference between the derivative model and the original model was 3 (1–5)%AS.

**Conclusion:**

We present a novel workflow to produce high-resolution patient-specific phantoms of coronary arteries.

## Introduction

The development and evaluation of cardiac catheterization devices, such as balloon catheters and coronary stents, rely on appropriate preclinical testing environments. Coronary artery disease (CAD) is a leading cause of morbidity and mortality and involves complex lesion as well as patient-specific anatomical variability that are difficult to replicate. While animal models provide a living biological system, they lack the pathological factor of human coronary arteries. Similarly, standard artificial models, such as those described in ASTM-F2394–07 (2022), often use idealized geometries that fail to account for the mechanical and anatomical variability observed in real patients. These limitations hinder the ability to assess device performance under relevant conditions. Therefore, there is a critical need for advanced coronary artery phantoms that accurately replicate human anatomy and pathology to support device development and procedural planning [1].

Despite the growing interest in 3D printed phantoms for cardiovascular applications, developing accurate and patient-specific coronary artery phantoms remains challenging. Most existing efforts have focused on larger cardiac structures such as the aorta, atria, or ventricles [2], while the complex and smaller-scale geometry of coronary arteries—with their tortuosity, branching patterns, and variable lesion morphologies—poses additional difficulties [3, 4]. Furthermore, lesion features such as calcifications or plaque burden are often inadequately represented in current models. The creation of high-fidelity phantoms depends on accurate 3D reconstructions from medical imaging. Coronary computed tomography angiography (CCTA) is currently the preferred imaging modality for 3D reconstruction and printing of heart phantoms [5] and coronary arteries [6]. However, the image resolution is insufficient to allow accurate reconstruction of the coronary arteries. Consequently, most models incorporate only the most proximal arterial segments [7]. Additionally, many reconstructions lack sufficient detail, especially in side branches or heavily calcified segments [8–10]. This is largely due to the more recent reliance on intravascular imaging, such as optical coherence tomography (OCT) or intravascular ultrasound (IVUS), fused with X-ray angiography, which has limitations in segmenting overlapping structures and detecting calcifications. These challenges indicate the need for improved 3D reconstruction approaches capable of capturing the full complexity of coronary anatomy.

To address these limitations, this study proposes a novel multi-modal image fusion approach that combines the high-resolution lumen detail of OCT with the broader anatomical context provided by CCTA. This integrated method enables the generation of regionally enhanced, high-fidelity 3D reconstructions that include side branches, calcifications, and patient-specific lesion characteristics. Using this approach, we have developed a library of anatomically accurate digital models that can be used for both virtual simulation and the fabrication of functional coronary phantoms. These models hold promise for improving the evaluation of catheter-based interventions and advancing the development of next-generation cardiovascular devices.

## Results

**Fig. 1:**
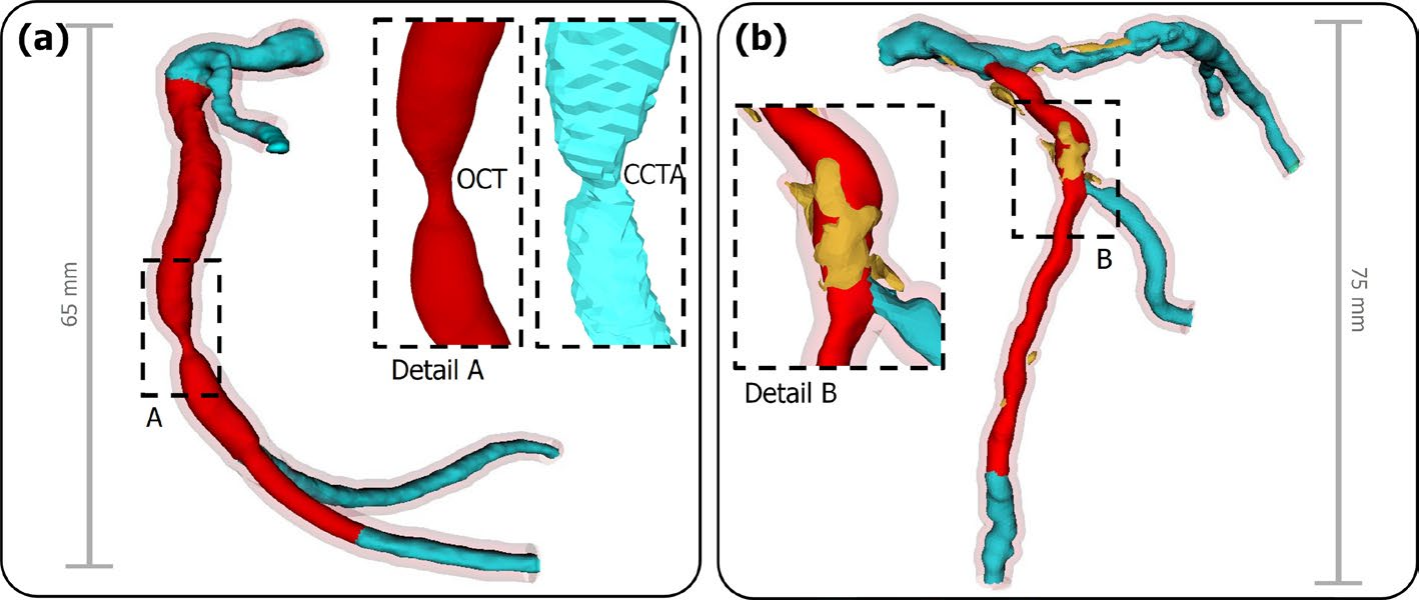
The red segment denotes data derived from OCT, while the blue segment corresponds to data obtained exclusively from CCTA. The reconstructed vessel wall is shown in transparent red. **a** Right coronary artery of patient 12 with a detailed view of the stenosis. **b** Left coronary artery of patient 7 with calcified plaque highlighted in yellow

In Fig. 1, we present a representative original model (OM) of both a left and a right coronary artery, based on the point-fuse methodology described in this study. The resulting models are shown as unified geometries, incorporating both OCT and CCTA data. In addition a detailed segment for the direct qualitative comparison of a digital model solely based on CCTA was added. A constant offset of the vessel lumen was used to reconstruct vessel wall. The presented OMs range between 65 mm and 75 mm in length, although the total length primarily depends on the segmented CCTA and can vary based on the specific region of interest. The right coronary OM of patient 12 did not include any calcifications, whereas patient 7 presented a significant amount of calcification, which correlates to the calcium score (CS) values shown in Appendix A Table 5.

### Adjusting calcifications

**Fig. 2:**
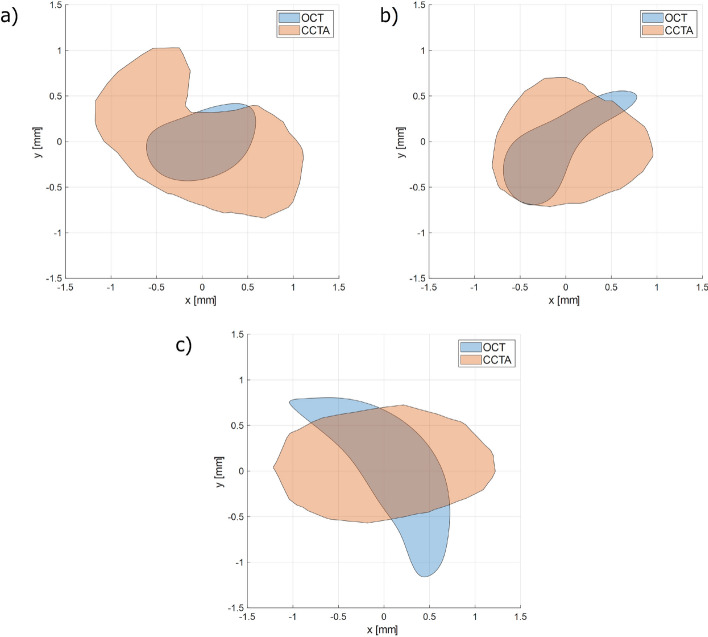
Superimposed calcium contours from CCTA and OCT illustrating selected frames for **a** patient 9, **b** patient 4, and **c** patient 7

For patients 4, 7, and 9, calcifications contours clearly visible on OCT were compared and superimposed with those identified on CCTA, as illustrated in Fig. 2.

This would lead to OCT and CCTA-based calcification parameters presented in Table 1.

**Table 1:** Calcification contour parameters based on OCT and CCTA

	OCT			CCTA		
Patient	Theoretical radius [mm]	Area [mm$^2$]	Perimeter [mm]	Theoretical radius [mm]	Area [mm$^2$]	Perimeter [mm]
7	0.68	1.46	5.77	0.86	2.34	5.95
9	0.49	0.74	3.26	0.90	2.52	6.81
4	0.49	0.75	4.13	0.76	1.81	4.97

### Patient and phantom data

We successfully enrolled 15 patients (26.6% female) in this study. A comprehensive list of all participants including sex, age, risk factor and lesion information as well as additional information on specific patients is provided in Appendix A Table 5. Table 2 shows the distances between side branches observed in patient OCT images and those acquired from the corresponding phantoms. Lesions with a range of area stenosis degree (%AS) from 45%AS up to 94%AS, and CS from 0 to 1161 were included. 11 participants exhibited greater than 75%AS, which also underwent percutaneous coronary intervention (PCI). Most of the significant lesions were located in the left coronary artery, with 10 in the proximal, mid, or distal segments of the left anterior descending artery, 1 in the first diagonal branch, and 1 in the left circumflex artery (LCx). Only 2 patients with significant lesions in the right coronary artery were included in this study. The median degree of stenosis determined for all patients was 80 (70–85)%AS. For the phantoms, the median degree of stenosis was 76 (72–80)%AS. The degree of stenosis for each phantom is also shown in Table 3. The resulting absolute median stenosis difference between the patient and the phantom was 3 (1–5)%AS.

**Table 2:** Distance between two side branches measured in the patient’s OCT and the OCT performed through the phantom

Patient	Sidebranches	Distance on patient-OCT [mm]	Distance on phantom-OCT [mm]
5	1	4	3
5	2	16	16
8	1	23	20
9	1	29	28

**Table 3:** Comparison of the original model (OM) and derivative model (DM), presenting the degree of stenosis (%AS) as well as the median and interquartile range (IQR; Q1–Q3) of vertex distance (VD) and area difference (AD) for each case

DM	%AS	VD [mm]	AD [mm2]
1	80	0.15 (0.09–0.22)	0.67 (0.13–1.67.)
2	60	0.18 (0.11–0.27)	1.21 (0.64–1.79)
3	74	0.17 (0.10–0.25)	0.77 (0.45–1.11)
4	–	–	–
5	76	0.14 (0.10–0.22)	0.76 (0.49–1.00)
6	–	–	–
7	80	0.14 (0.09–0.22)	0.61 (0.30–1.03)
8	39	0.16 (0.10–0.25)	0.96 (0.37–1.51)
9	82	0.14 (0.09–0.20)	0.75 (0.42–0.98)
10	74	0.14 (0.10–0.23)	0.96 (0.65–1.59)
11	75	0.14 (0.09–0.23.)	0.69 (0.42–1.20)
12	89	0.24 (0.13–0.38)	2.42 (0.66–3.71)
13	80	0.18 (0.12–0.27)	1.49 (1.00–2.14)
14	84	0.15 (0.10–0.22)	0.40 (0.23–0.75)
15	68	0.14 (0.09–0.23)	0.75 (0.55–0.98)
Median	76 (72–80)	0.15 (0.14–0.17)	0.76 (0.69–1.02)

**Fig. 3:**
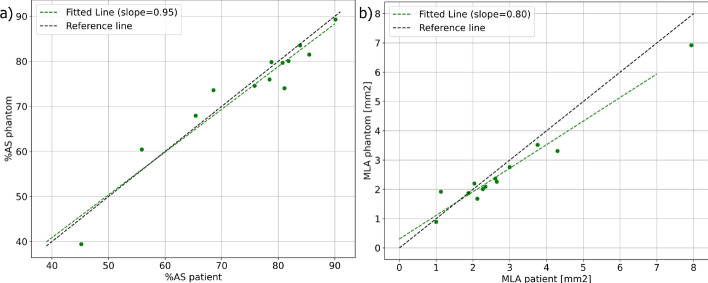
Linear regression analysis comparing **a** the OCT-derived area stenosis (%AS) and **b** minimum lumen area (MLA) determined for each patient and corresponding phantom

Linear regression analysis of the area stenosis yielded a slope of 0.95 with an R$^2$ value of 0.92. Similarly, linear regression analysis of the minimum lumen area (MLA) produced a slope of 0.80 with an R$^2$ value of 0.96. Those results are also shown in Fig. 3.

### Validation and accuracy of the workflow and phantoms

**Fig. 4:**
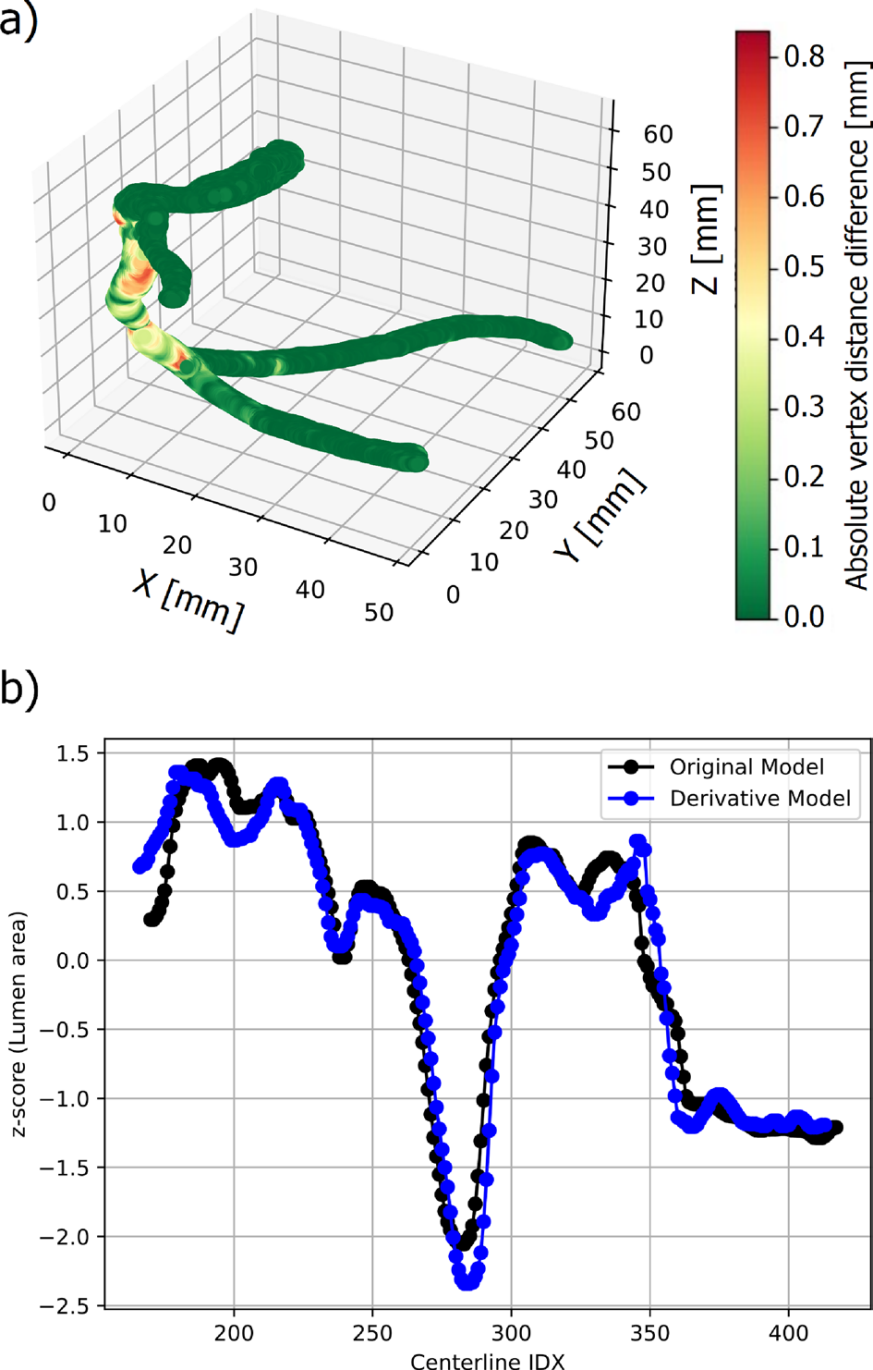
**a** Superimposed original model (OM) and derivative model (DM) of patient 12 including heatmap to indicate vertex distance (VD) between the two models. **b** z-score normalized lumen area comparison

Fig. 4 illustrates the vertex-wise deviations (in mm) between the OM and derivative model (DM) of patient 12. For this case, the maximum deviation was 0.84 mm, located at the proximal segment of the reconstructed coronary artery.

The median vertex difference (VD) between OM and DM was 0.24 (0.13–0.38) mm. Comparable analyses, including superimposed visualizations, were performed for all validated cases. The normalized area, expressed as the z-score at each centerline point for patient 12, is also shown in Fig. 4. The mean z-score difference in this example was 0.37±0.37. Across all validated cases, the median VD was 0.15 (0.14–0.17) mm, while the median area difference (AD) between OMs and DMs was 0.76 (0.69–1.00) mm$^2$. The median R$^2$ value for the z-score correlation was 0.93 (0.91$-$0.94). A summary of the results for all 13 patients, including the median VD and AD, is provided in Table 3.

## Discussion

In this study, we present ultra-high resolution patient-specific coronary artery models, based on photon-counting CCTA and OCT. Our data fusion methodology combines the strengths of both modalities, capturing the detailed features and vessel morphology from OCT while incorporating the 3D anatomical information (including centerlines and side branches) from CCTA. This approach overcomes resolution limitations and enables reconstruction of distal coronary segments [7]. The resulting models provide a valuable foundation for device testing and the development of functional phantoms using additive manufacturing.

### Evaluation of 3D models and phantoms

With the demonstrated workflow, reliable and highly accurate 3D models of the coronary arteries can be generated from OCT pullback and CCTA images. These models include calcifications and side branches. Although we did not systematically record processing times, we observed that creating the model typically took operators around 20 min. This informal observation suggests a potential improvement over the processing times reported by Wu et al., which were approximately 56 min [11]. The comparison of distances between side branches measured on the patient OCT images and the OCT images acquired through the corresponding phantoms demonstrated good spatial agreement. The observed deviations (up to 3 mm) can be attributed to minor registration inaccuracies, slight deformations introduced during phantom fabrication, or catheter movement related to myocardial contraction in the patient. CCTA-derived calcification contours exhibited systematically larger dimensions than those obtained from OCT. Across the analyzed cases, CCTA-based theoretical radii exceeded OCT values by approximately 0.2–0.4 mm. To mitigate this systematic overestimation, a uniform offset of 0.5 mm was applied to the CCTA-derived contours. This adjustment falls within the expected spatial uncertainty of CCTA and provides a pragmatic correction to improve geometric correspondence with the higher-resolution OCT reference. The workflow could successfully be validated using the 3D printed phantoms and create DMs. The median vertex deviation observed in this study was low (0.15 (0.14$-$0.17) mm), reflecting the improved geometric accuracy achieved through the integration of high-resolution OCT data. In contrast, CCTA alone has a spatial resolution of approximately 0.4 mm, which would inherently limit the precision of anatomical reconstruction and result in greater deviations. Although absolute differences between models were present, these deviations remained below the resolution threshold of CCTA. This suggests that the reconstruction process-including imaging, modeling, and fabrication-preserves anatomical detail with sufficient accuracy for applications such as catheter navigation and device testing. The largest vertex deviations occurred at the transitions between the CCTA and OCT data, specifically at the start and end of the pullback images and at each bifurcation, as indicated in Fig. 4. This issue arises from the semi-automatic data fusion process, where overlapping CCTA and OCT points are removed. Since CCTA has a significantly lower resolution than OCT, adjustments should prioritize the higher-resolution modality (OCT). A possible approach to overcome this issue would include the adjustment and resampling of the CCTA data points to fit the start and end of the OCT pullback data points. Further, to increase the accuracy at bifurcations, attempts can be made to segment the bifurcations on the pullback images. However, the frame rate during the pullback might be too low to accurately delineate the side branch. Another limitation during OCT pullback is the influence of the cardiac cycle, which can result in lumen images being captured at different phases. ECG-triggered OCT could potentially mitigate this effect. Additionally, lateral movement between the catheter and vessel wall during cardiac contraction is suspected, though this could not be accounted for in the current study. In our dataset, the median area difference between OM and DM was 0.76 (0.69–1.02) mm$^2$. This difference reflects systematic errors associated with OCT acquisition and 3D printing, which are also indicated in the results of the linear regression analysis of the MLA. Therefore, it makes sense to normalize the data by using the z-score. The z-score allows for a standardized, scale-independent comparison of the relative sizes of the lumen area across the OM and DM. The difference of DM and OM produced a median z-score difference of 0.22 (0.18$-$0.27). This implies that the two models, on average, had similar deviation from their respective means. The observed differences as shown in Fig. 4 can be caused by a registration error indicating a variable inter-frame distance. It is also possible that the area differences occur due to variations during the recording process, such as catheter positioning, heart movement, or recording medium. Many previous studies have validated the accuracy of their lumen segmentation but have not assessed the accuracy of their 3D reconstructed models [12, 13]. We compared our results with the few studies that did and successfully introduced additional features, achieving comparable or improved accuracy, as shown in Table 4.

**Table 4:** Comparison of previous studies on the inclusion of calcifications (C), side branches (SB), validation (Val), and accuracy metrics, including the mean R$^2$ value of the linear regression of lumen areas and the mean vertex distance (VD)

Author	C	SB	Val	R$^2$	VD [mm]
Wu [11]	-	+	+	0.95 (0.93–0.96.93.96)	-
Solanki [10]	-	+	+	-	0.44 (0.34–0.61)
Papafaklis [14]	-	-	-	-	-
Bourantas [15]	+	-	+	-	0.70±0.03
This work	+	+	+	0.93 (0.91–0.94.91.94)	0.15 (0.14–0.17)

### Outlook

Future computational models and physical phantoms should incorporate not only anatomically accurate vascular representations, but also functional properties, such as vessel compliance (i.e., deformation under physiological pressures) and both elastic and plastic deformations induced by PCI, including stent deployment and balloon angioplasty. In addition to accurately modeling the vessel lumen, it is crucial to account for wall thickness and calcifications. One limitation of the present study lies in the assumption of a uniform vessel wall thickness of 1 mm and the neglect of detailed vessel wall layers and plaque pathologies beyond calcification. Recent advances in deep learning–based methods have demonstrated the potential to capture vessel lumen, tissue components, and possibly vessel wall characteristics, thereby enabling more precise segmentation of OCT images, as reported in previous work [16, 17]. This, in turn, may lead to further quality improvements by facilitating the extraction of critical features from high-resolution OCT images. If these features are incorporated into the 3D models, compliant multimaterial phantoms can be fabricated using PolyJet 3D printing technology [18]. Additionally, different applications of the presented models can be envisioned, such as cardiac device testing or the optimization of CCTA protocols as suggested in [1] and [19]. After manufacturing, these applications will require thorough validation. To improve the presented workflow in the future and particularly once a larger number of models are available, a deep learning–based registration approach could be developed to automate the process. Such methods, however, require reliable training data, which would initially be based on different operators. Therefore, a thorough user variability study would still be essential to ensure the robustness and generalizability of any automated approach.

## Conclusion

We developed and validated a semi-automatic workflow that utilizes clinical data for accurate digital reconstruction and 3D printing of coronary arteries. By combining OCT and CCTA, leveraging their complementary strengths, we achieved patient-specific models with unprecedented precision (absolute vertex differences of 0.15 mm, stenosis differences of 3%). This novel workflow, complemented by clinical data, demonstrates significant potential for advancing patient-specific testing environments.

## Methods

### Clinical data

We performed a clinical trial to acquire a dataset including ultra-high resolution images (i.e., photon-counting CCTA and OCT) of healthy and diseased coronary arteries. The study was approved by the local ethics committee in Bern, Switzerland (project ID 2023–00,666). The study enrolled 15 patients for imaging and data collection. Informed consent was obtained from all participants. All methods were carried out according to the relevant guidelines and regulations. Patients were included if they had a clinical indication for coronary angiography and a prior CCTA examination showing significant stenosis before the angiography. In general, individuals with acute coronary syndrome, congestive heart failure, or atrial fibrillation were excluded. We created a patient vignette that included sex, age, and risk factors such as arterial hypertension (HTN), family history of coronary artery disease (FH), and diabetes (D). All CCTAs were acquired using a NAEOTOM Alpha photon-counting CT scanner (Siemens Healthineers, Germany), with a slice thickness and pixel spacing of 0.4 mm. The CS (or Agatston score [20]) was calculated from the CCTA for each patient in the most severely diseased vessel, which was later imaged using OCT. Additionally, the degree of stenosis (%Sten) identified on the CCTA was recorded for the vessel that was later treated. All participants underwent a scheduled coronary angiography, with subsequent OCT-guided PCI performed selectively based on the invasive findings.

### Invasive measurements

During the minimally invasive procedure performed by an interventional cardiologist, the most severely diseased vessel lumen was identified and recorded at the beginning of the procedure using an OCT imaging catheter (Dragonfly OpStar™ Imaging Catheter, Abbott, USA). Dynamic OCTs were performed with a pullback speed of 40 mm/s over a distance of 75 mm. However, the effective imaging length is typically slightly shorter, since frames at the start or end of the pullback may contain blood artifacts that preclude reliable lumen contour assessment. The lumen contours were semi-automatically segmented from the images acquired during the OCT pullback using the AptiVue™ software by Abbott, USA. The software allowed for automatic identification of lumen border. The MLA and a reference vessel area (RVA) were determined. The RVA was measured proximal to the MLA in a healthier segment of the vessel to determine the OCT-derived %AS as defined by (1):

1 $$\begin{aligned} \%AS = \frac{RVA-MLA}{RVA} \times 100. \end{aligned}$$

### Generating 3D models

OCT was utilized to accurately define the inner vessel wall. CCTA data were used to determine the precise vessel pathways. Using CCTA data, the three-dimensional structure and centerline of the coronary arteries were reconstructed. A custom workflow, described in the following sections, was developed to efficiently and reliably generate high-resolution models of the coronary arteries by integrating data from the OCT and CCTA images. For clarity and readability, we defined three types of models:

- Original model (OM): the digital model generated using the custom workflow based on patient-acquired data.
- Phantom: a 3D-printed physical model derived from the original model.
- Derivative model (DM): the digital model generated using the custom workflow based on data acquired from the phantom.

**Fig. 5:**
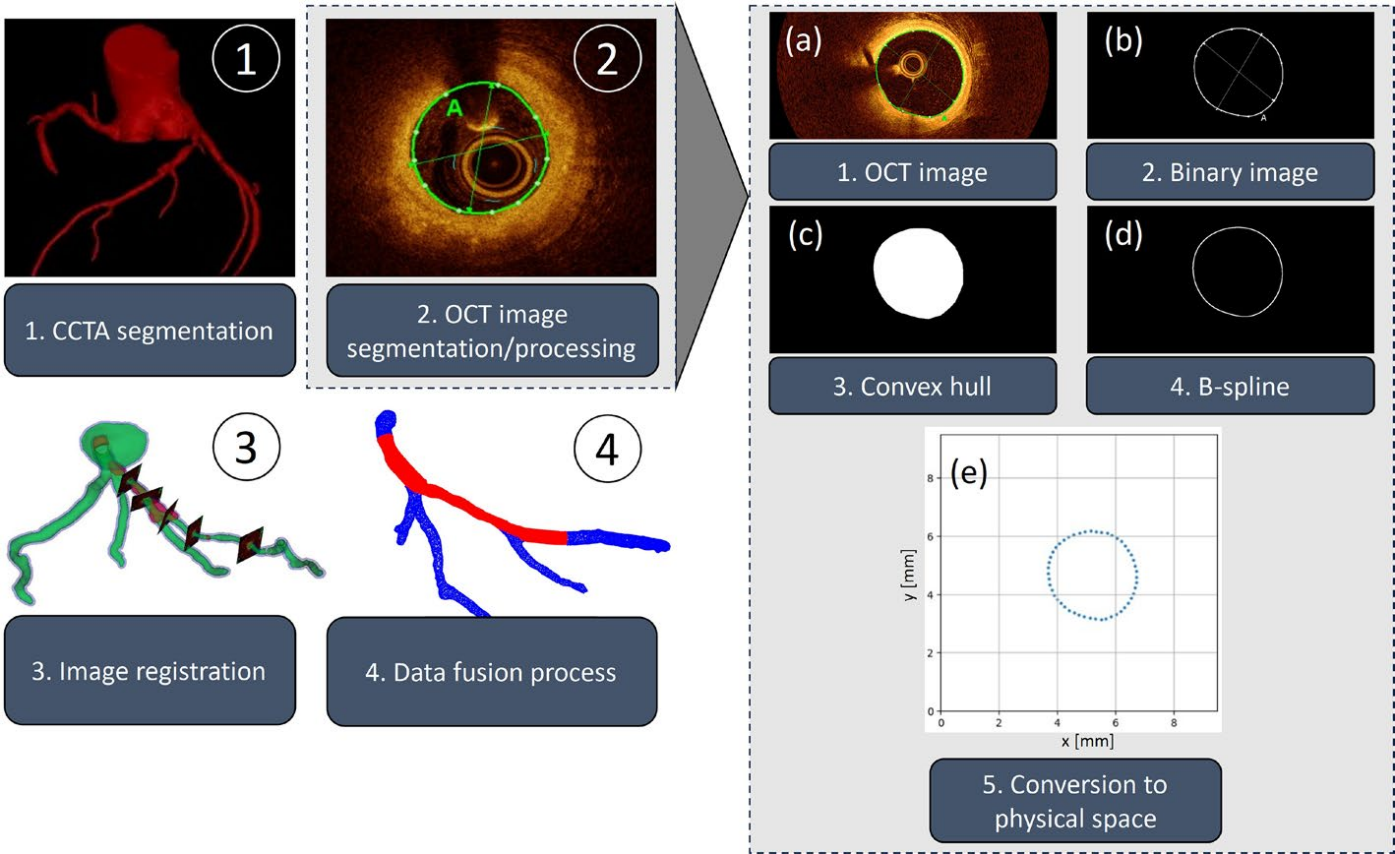
Workflow for generating high-resolution 3D models of coronary arteries (left) and processing of OCT images to obtain vessel contours (right): **a** semi-automatic lumen delineation, **b** binary mask generation, **c** convex hull application, **d** B-spline fitting, and **e** conversion from pixel to physical space

**Fig. 6:**
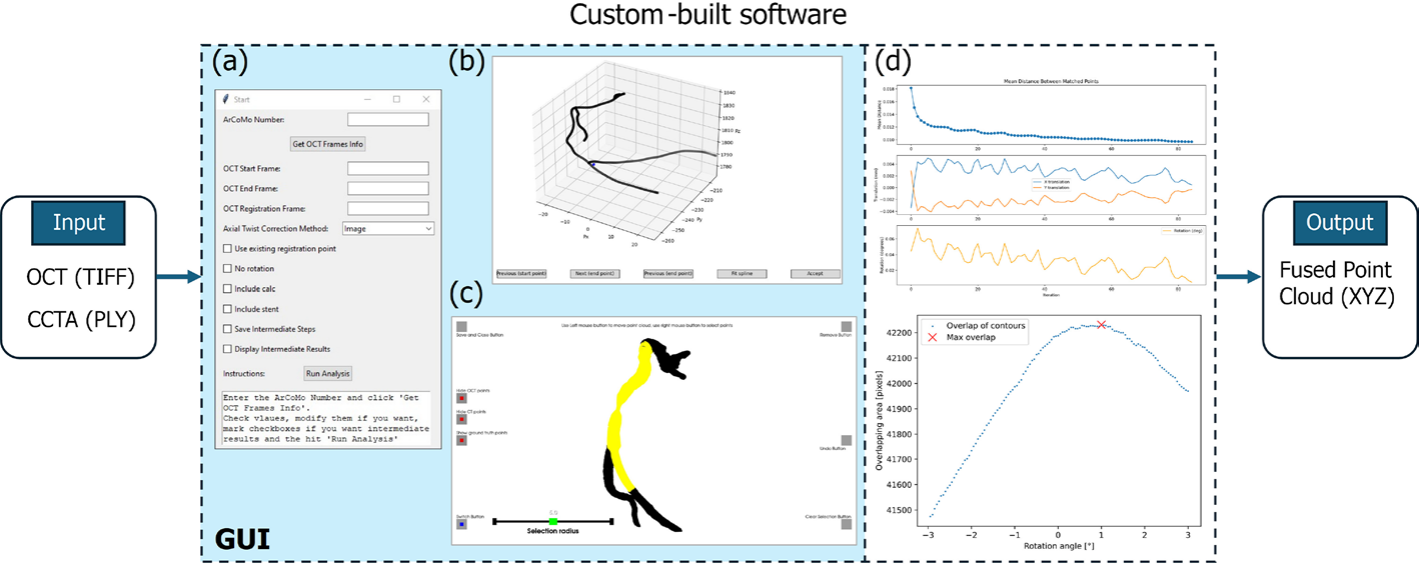
Custom-built software for the fusion of OCT and CCTA images, featuring a GUI. **a** Main dialog box for selecting processing parameters and providing user instructions. **b** Toolset for centerline adjustment. **c** Visualization and manipulation of the fused OCT-CCTA data. **d** Optional transitional outputs to verify intermediate results and data alignment

The workflow can be separated into four individual steps: CCTA image segmentation, OCT image processing and segmentation, the alignment of the OCT lumen on the CCTA centerline (image registration), and the data fusion process (Fig. 5). For the segmentation of both OCT and CCTA images, various commercial and open-source software solutions are available and their pre-processed image data could be used as input data for our developed software. Processing, image registration as well as the core feature enabling the actual high-resolution data fusion process was based on a novel custom-built software including its own graphical user interface (GUI), which is also illustrated in Fig. 6. Further details on the algorithms and methodology used in each step are described below.

#### CCTA segmentation

The coronary artery lumen and calcifications were segmented in the CCTA images using Mimics Innovation Suite (Mimics and 3-matic) software package (Materialize, Belgium). First, the coronary lumen and calcifications were identified using the cardiovascular tool within the software. Then, manual corrections were made to the contour details. The centerline of the segmented lumen was derived using integrated algorithms that incorporated skeletonization, graph-based path finding, and smoothing procedures, allowing for an accurate representation of the vascular structures. The centerline was exported as a point-cloud text file. The segmented lumen was exported as polygon file storing the 3D information.

#### OCT image segmentation/processing

The complete processing of the OCT pullback images is schematically visualized in Fig. 5(a-e). The previously identified lumen border of every frame was visually examined and manually corrected if needed to ensure accurate delineation as shown in Fig. 5(a) before exporting it as tagged image file format (TIFF). The exported image would include both, the image data as well as the delineated lumen border. The images had a resolution of 1024 x 1024 pixels, with 103 pixels corresponding to 1 mm. The images were further processed to extract the lumen contour using a custom-built software, which performed the following steps. At first the images were filtered to isolate the lumen border markings using color thresholding, which creates a binary mask (Fig. 5(b)). To find the exact lumen border, noise and artifacts were removed using a Gaussian kernel followed by intensity threshold filtering. To obtain a closed mask, a convex hull was applied, representing the smallest convex polygon enclosing all points on the contours (Fig. 5(c)). A B-spline representation was then identified around the mask to find a smooth approximation of the 2D lumen contour (Fig. 5(d)). Data points from the spline were sampled and converted from image space (discrete pixel space) to the physical space in millimeters (Fig. 5(e)). This process was automatically performed for each frame of the recorded pullback image.

#### Image registration

Prior to registration, the axial twist of the catheter during pullback must be corrected. To determine the corrective rotation angle required to compensate for this twist and to ensure proper alignment of consecutive OCT contours, we implemented and evaluated several alignment algorithms, including normalized image cross-correlation, the iterative closest point (ICP) method, and an area-overlap optimization approach. The final 3D models generated for 3D printing of the phantom, we selected the area-overlap optimizer due to its superior robustness and accuracy. As described in previous work by Wu et al., the algorithm identifies the optimal alignment by rotating each pair of consecutive contours within a range of $-3^{\circ }$ to $+3^{\circ }$ and computing the overlapping area at each step [11]. The angle yielding the maximum overlap was then selected as the corrective rotation angle. In order to create a 3D structure out of the 2D lumen contours obtained during the OCT processing a registration of the images is necessary. The CCTA imaging data acted as target for the movable OCT data. The OCT image slices were going to be stacked along the extracted centerline. Typically bifurcations, which were visible in both the OCT and the CCTA, were chosen as anatomical landmarks for alignment.

The landmark was manually selected at the carina point of the bifurcation on a single OCT frame. In the CCTA, the corresponding landmark was selected using the reconstructed centerline. The actual registration point on the CCTA was defined in our custom software as the midpoint between the manually selected projection points on the main branch and the side branch vessels (Appendix B, Fig. 11). This midpoint was matched with the carina point on the OCT frame by applying translation and rotation to the corresponding 2D lumen contour. The remaining 2D lumen contours were then aligned with the centerline points before and after the registration point. Out-of-plane rotations were not considered, as the catheter path was assumed to approximately follow the vessel centerline, particularly in small vessels. This registration approach and the underlying assumption were evaluated in a validation experiment, which is described in Appendix C. The centerline was resampled to ensure that the distance between points matched the distance between OCT frames. Interlocking the 2D lumen contours ensured that the relative position of each frame remained unchanged. After the alignment of the lumen contours, which were now positioned in the 3D space, each contour was again rotated to be orthogonal to the centerline.

#### Adjusting calcifications

**Fig. 7:**
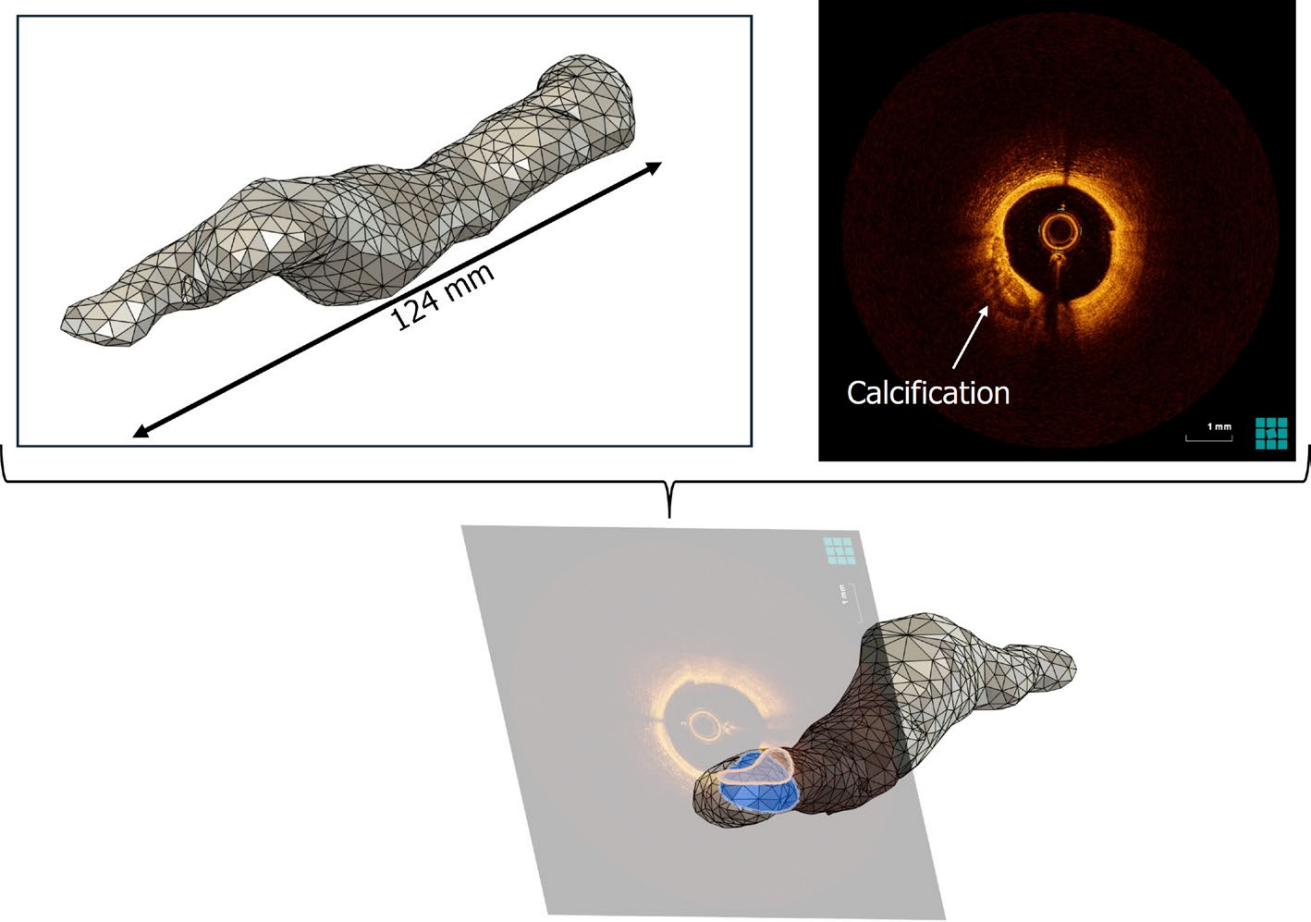
The 2D OCT frame displaying clearly defined calcification borders was registered to the 3D calcification model reconstructed from the CCTA. The intersection of the two datasets was used to generate a 2D cross-section of the CCTA-derived calcification

Segmented calcifications from the CCTA data were incorporated into the digital models. Since calcifications on CCTA are often overestimated due to the blooming effect, corresponding OCT images were used to identify an appropriate correction factor. Specifically, frames in which calcification borders were clearly visible on OCT were selected and compared with the calcifications detected on CCTA at the same anatomical location. The spatial transformation previously applied to the lumen was also applied to the calcifications to ensure alignment between modalities. A plane intersecting both the OCT-derived and CCTA-derived calcifications was used to generate comparable cross-sectional contours. The resulting calcified contours were exported and analyzed with respect to their theoretical radius, area, and perimeter. The concept is also visualized in Fig. 7.

#### Data fusion

**Fig. 8:**
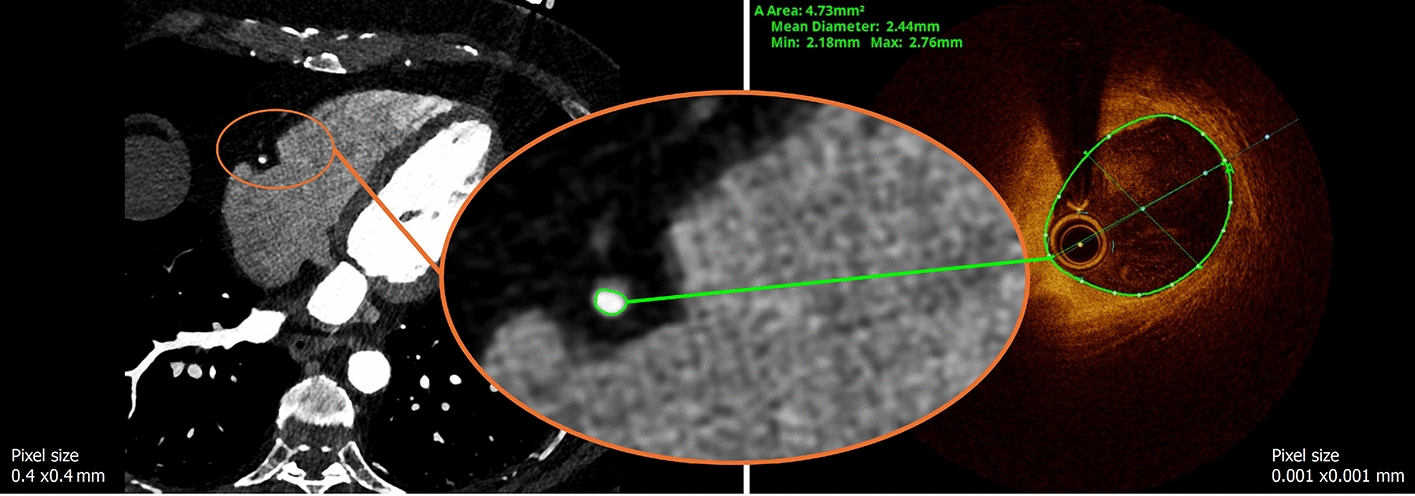
Comparison of CCTA (left) and OCT (right) imaging of the lumen in the right coronary artery of patient 12. OCT provides higher resolution and clearer delineation of the lumen boundary compared to CCTA

*Concept* The general idea of data fusion in this context is to combine information from both OCT and CCTA to improve the accuracy of 3D model reconstruction. CCTA provides essential spatial information, including the vessel centerline and side branches, while OCT offers highly detailed lumen representations. With a spatial resolution approximately 400 times greater than that of CCTA, OCT significantly enhances the level of detail in 3D reconstructions. This fusion process particularly benefits from OCT’s precise depiction of the lumen. As illustrated in Fig. 8, the lumen boundary is clearly defined in OCT images, whereas it appears less distinct and more challenging to identify accurately in CCTA data.

**Fig. 9:**
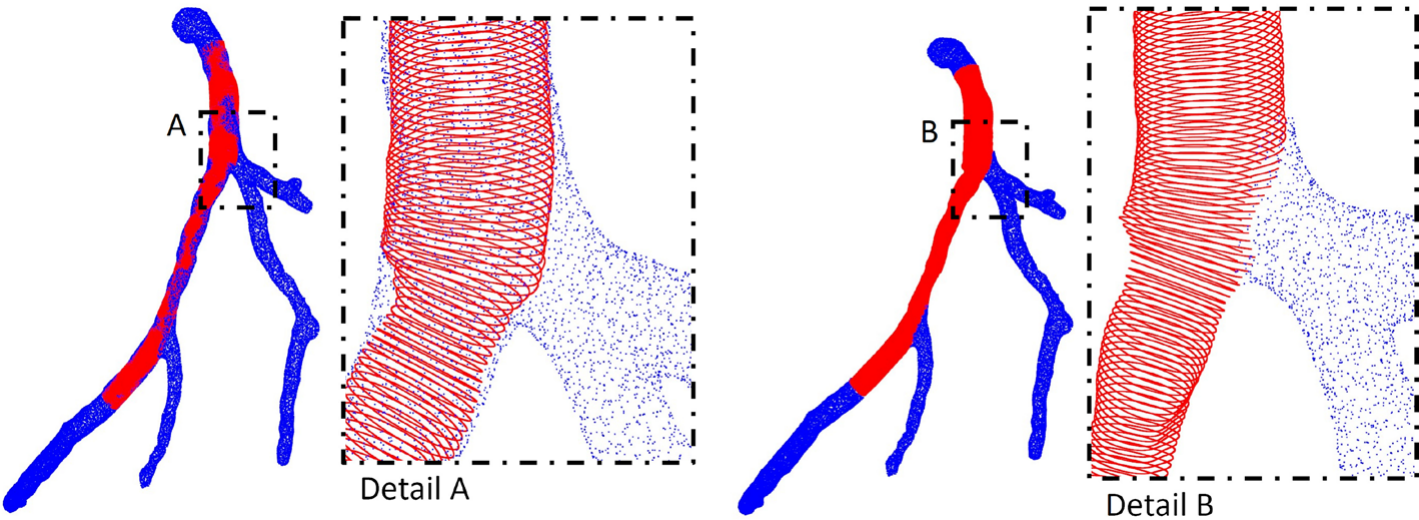
Merged point clouds of the CCTA (blue) and OCT (red) before (left) and after (right) removal of overlapping points

*Process* A 3D point cloud was extracted from the polygon mesh obtained from the CCTA. This CCTA point cloud was then merged with the transformed point cloud derived from the OCT images. The merged point cloud contained the lumen geometry of side branches and segments both proximal and distal to the OCT segment, as well as the OCT-covered segment itself. We refer to this process as data fusion. Because the centerline was based on the same CCTA data, no additional registration was required. However, the CCTA point cloud overlapped with the lumen contours extracted from the OCT images. To resolve this, we developed a semi-automatic procedure in a custom graphical user interface (GUI) that removes the overlapping CCTA points. The automatic removal includes the detection of points on the CCTA with close distance (<0.35 mm) to OCT point cloud using k-d trees and finding nearest neighbors within a certain radius. Afterwards residual points are being removed using a point selection tool via our custom GUI enabling the selection of different regions and points, resulting in the final fused point cloud shown in Fig. 9.

The fully processed points representing the lumen information from both images were meshed using the MeshLab open-source system (v2023.12) [21] to create a 2D shell. Using the 3-matic software (v17.0, Materialize, Belgium), a 3D solid by adding calcifications and wall thickness was created. The previously segmented calcifications were scaled and modified based on the outcomes of the mentioned adjustment process to account for the blooming effects. The final OM featured a uniform wall thickness of 1 mm, as previously reported [22], except in regions with calcifications, where the calcifications’ thickness must be added to determine the total wall thickness.

### Validation of the 3D models

To assess the robustness and precision of our reconstruction workflow, we compared the DMs, generated from re-imaging the printed phantom, to the OMs. This comparison provides insight into the cumulative geometric deviations introduced by each step in the modeling and fabrication process. 15 patient-specific OMs were created for the validation process. To validate the steps 2, 3, and 4 described above and shown in Fig. 5, establishing a ground truth was essential. Since the actual geometry of the patient’s coronary arteries cannot be used directly as these are vital organs, we decided to use the OM created for each patient in this study as the ground truth.

**Fig. 10:**
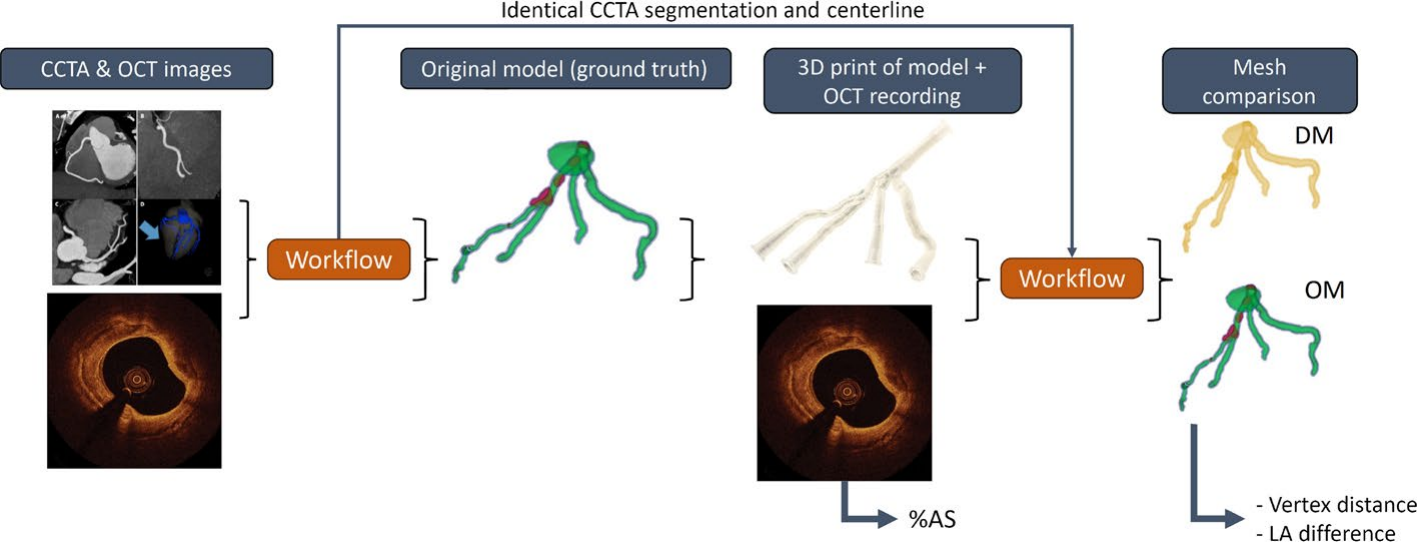
Schematic illustration of the validation process: generation of a DM followed by superimposition of the OM and DM to evaluate vertex distance and lumen area

Several initial prototypes were manufactured via PolyJet 3D printing technology on a Stratasys Inc., USA, J750 Digital Anatomy 3D Printer (DAP), yielding a compliant coronary artery phantom with a resolution of 42$\times$42$\times$14 $\mu$m. Additionally, rigid phantoms were produced using an Anycubic Photon M3 Max, a stereolithography (SLA) based printer achieving a resolution of 50$\times$50$\times$50 $\mu$m. The predefined printing parameters for standard resins were utilized. The comparison between the OM and DM is not intended to evaluate the physical accuracy of the 3D-printed phantom, but rather to assess the overall accuracy and consistency of the reconstruction process, from initial imaging through segmentation, 3D modeling, fabrication, and re-imaging. By quantifying the geometric deviations between OM and DM.

After printing, an OCT pullback of the 3D printed phantom through the segments, that were initially recorded in the clinical setting, was performed in a water bath using a Dragonfly OpStar™ Imaging Catheter (Abbott, USA). The images were processed using the previously described workflow and merged with the patients CCTA data, leading to a DM. Additionally, the %AS of the DM and the MLA were determined in a manner similar to the method previously used on the patient’s OCT image. Both %AS and MLA were assessed using a linear regression analysis. In three selected patients presenting more than one prominent side branch visible on OCT, the inter-branch distances were measured on the original patient OCT datasets as well as on the OCT images acquired from the corresponding phantoms. Subsequently, the OM was compared to the DM. Two different approaches were used to assess the accuracy:

- For one approach the point cloud of the DM was superimposed onto the point cloud of the OM in MeshLab. The distance of each vertex between the OM and the OM was calculated, resulting in a heatmap where large distances indicate large deviations, and vice versa. Furthermore, the median vertex distance and the corresponding interquartile range (IQR) were determined for each validated model. Absolute vertex distances smaller than 0.05 mm were excluded to avoid bias from negligible meshing differences, which may also result from identical points originating from the CCTA mesh. This approach is conservative, as eliminating small errors tends to increase the resulting median distance.
- The second validation approach would assess the lumen area at each centerline point for both the OM and DM. To determine the lumen area a plane perpendicular to the centerline was generated for each point on the centerline. The lumen was defined by the area enclosed by the contour of the digital model intersecting with the generated plane. The absolute AD was assessed. Additionally, the lumen areas were normalized using the z-score (A-$\mu$)/$\sigma$ with $\mu$ referring to the mean area, and $\sigma$ the standard deviation of the mean. The z-score accounts for systematic dimensional discrepancies between the OM and DM caused by 3D printing or OCT imaging. From there the z-score difference for each validated case could be evaluated. Additionally, the correlation of the z-scores determined for the OM and the DM was assessed using a linear regression analysis. $R^2$ values were determined for each correlation.

The validation process is also conceptually illustrated in Fig. 10.

## Figures and Tables

**Fig. 11:**
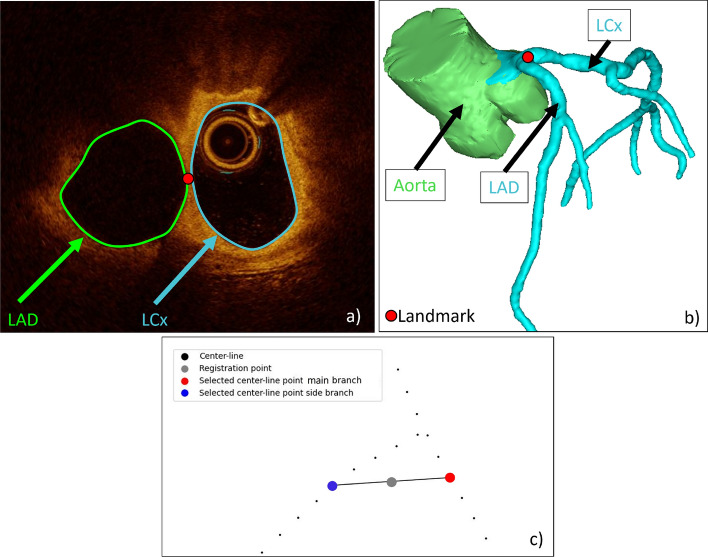
Conceptual registration between OCT and CCTA using the carina point of a bifurcation, identifiable in both **a** the OCT image and **b** the CCTA. **c** In our software, the projections of the registration point onto the main and side branches were manually selected and adjusted within the graphical user interface. These projections were then used to compute the actual carina point and achieve precise alignment between OCT and CCTA

**Fig. 12:**
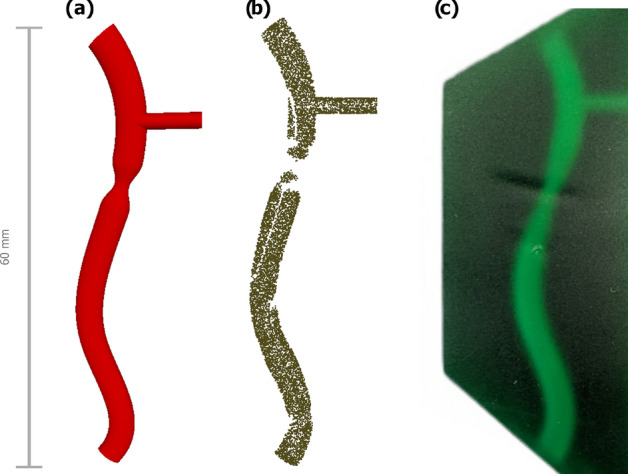
**a** Known ground truth of a simplified coronary artery model. **b** A reduced point cloud with spatial resolution similar to photon-counting CCTA. **c** 3D printed negative of the lumen

**Fig. 13:**
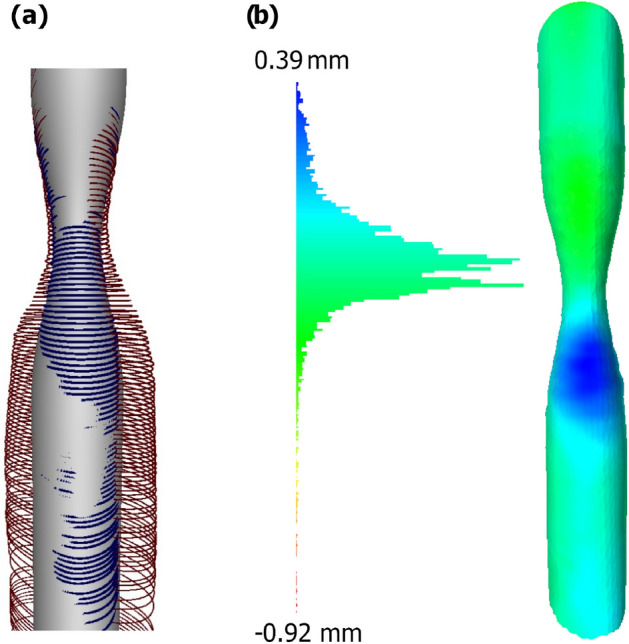
**a** Axial rotation correction—ground truth mesh of the self-designed model in grey with correctly rotated OCT lumen contours (blue) and with the original OCT lumen contours (red). **b** Superimposed OCT segment and ground truth of the simplified coronary artery including heatmap to indicate vertex distance (VD) between the two models

## Data Availability

The data that support the findings of this study are not publicly available due to institutional and privacy considerations. Selected data may be made available from the corresponding author upon reasonable request.

## Appendix A: Patient data

See Table 5.

**Table 5:** Overview of participants and parameters identified in this study

Patient	Sex	Age	Hypertens.	Fam Hist.	Diabet.	Lesion	MLA	% AS	% Sten	Calc. Score
1	M	59	+	+	-	Mid LAD	2.1	82	20-50	265
2	M	63	-	-	+	Prox LCx	7.9	56	50	217
3	F	76	+	-	-	Mid LAD	1.1	81	70-90	274
4	F	64	-	-	+	Mid LAD	0.7	91	50-70	473
5	F	70	+	-	+	D1 LAD	1.9	79	70-90	142
6	M	59	+	-	-	Dist LCx	0.7	94	90-99	182
7	M	67	+	-	+	Mid LAD	2.3	79	50-70	1161
8	F	63	+	-	-	Mid LAD	3.8	45	50-70	99
9	M	70	-	-	-	Mid LAD	2.6	86	70-90	331
10	M	60	+	+	+	Prox LAD	3.0	69	50-70	28
11	M	62	+	-	+	Mid LAD	2.3	76	50-70	122
12	M	62	-	-	-	Mid RCA	1.0	90	70-90	0
13	M	60	+	+	-	Dist RCA	2.7	81	70-90	213
14	M	39	+	-	+	Mid LAD	2.0	84	n/a	0
15	M	73	+	-	-	Mid LAD	4.3	65	50-70	716

Hypertension, family history of coronary artery disease, diabetes, minimum lumen area (MLA) [mm2], OCT-derived degree of stenosis (%AS), CCTA-derived degree of stenosis (%Sten), and calcium score

A comprehensive list of all participants is presented in Table 5. Patient 14 exhibited 90–99%Sten in the LCx artery, leading to a referral for invasive assessment and inclusion in this study. However, the invasive assessment revealed no clinically significant stenosis in the LCx but identified a significant lesion in the LAD, which was reconstructed and used for the study.

## Appendix B: Registration landmark

See Fig 11.

## Appendix C: Validation experiment for registration workflow

The aim of this experiment was to validate the reconstruction methodology under controlled conditions, ensuring that any observed deviations were attributable to the algorithm itself rather than to acquisition-related factors. Consequently, the simulated CTCTA represents an idealized scenario. A simplified coronary artery model was created using computer-aided design (CAD) software, which served as the ground truth geometry. From this model, a point cloud with a spatial resolution of 0.4 mm (comparable to photon-counting CCTA resolution) was generated to simulate CCTA data. The CAD model was fabricated using stereolithography (SLA) 3D printing. The different models and prints are shown in Fig. 12

Optical coherence tomography (OCT) imaging was then performed on the printed phantom, and the acquired data were processed using the same workflow as for our clinical cases. The resulting OCT-based reconstruction was compared to the CAD ground truth model. The evaluation showed a median vertex difference of 0.11 (0.08–0.15) mm. Qualitative inspection further demonstrated accurate alignment of the OCT contours along the original model geometry. Figure 13 illustrates the comparison between the OCT-based model and the CAD ground truth, including the OCT contours before and after axial twist correction.These findings support the validity of our assumption and demonstrate that the proposed registration workflow provides accurate and reliable results in the studied vessel regions.
